# Outbreak of SARS-CoV-2 at a hospice: terminated after the implementation of enhanced aerosol infection control measures

**DOI:** 10.1098/rsfs.2021.0066

**Published:** 2022-02-11

**Authors:** Luke Feathers, Tracey Hinde, Tammy Bale, Jo Hyde, Paul W. Bird, Christopher W. Holmes, Julian W. Tang

**Affiliations:** ^1^ LOROS Hospice Care, Leicester, UK; ^2^ Clinical Microbiology, University of Leicester Hospitals, 5/F Sandringham Building, Leicester Royal Infirmary, Infirmary Square, Leicester LE1 5WW, UK; ^3^ Respiratory Sciences, University of Leicester, Leicester, UK

**Keywords:** hospice, outbreak, COVID-19, aerosol, nosocomial, transmission

## Abstract

Outbreaks of COVID-19 in hospices for palliative care patients pose a unique and difficult situation. Staff, relatives and patients may be possible sources and recipients of infection. We present an outbreak of COVID-19 in a hospice setting, during the UK's first pandemic wave. During the outbreak period, 26 patients and 30 staff tested SARS-CoV-2 positive by laboratory-based RT-PCR testing. Most infected staff exhibited some mild, non-specific symptoms so affected staff members may not have voluntarily self-isolated or had themselves tested on this basis. Similarly, for infected patients, most became symptomatic and were then isolated. Additional, enhanced aerosol infection control measures were implemented, including opening of all windows where available; universal masking for all staff, including in non-clinical areas and taking breaks separately; screening for asymptomatic infection among staff and patients, with appropriate isolation (at home for staff) if infected; performing a ventilation survey of the hospice facility. After these measures were instigated, the numbers of COVID-19 cases decreased to zero over the following three weeks. This outbreak study demonstrated that an accurate understanding of the routes of infection for a new pathogen, as well as the nature of symptomatic versus asymptomatic infection and transmission, is crucial for controlling its spread.

## 1. Background

The COVID-19 pandemic has been ongoing since January 2020 with multiple outbreaks reported in various healthcare [1] and non-healthcare settings, including public transport [2,3], workplaces [4], schools [5,6], churches [7,8], recreational [9,10] and entertainment venues [11,12].

The various modes of transmission of SARS-CoV-2 have been investigated and debated in the context of infection control, to optimize the interventions used to reduce the spread of this virus [13–15]. Currently, the main route of transmission is considered to be predominantly via short- and long-range aerosols, especially in crowded, poorly ventilated, indoor spaces, with direct (via touch) and indirect contact (via contaminated fomites) playing a relatively minor role [16,17].

Healthcare settings, particularly long-stay residential homes, have been hardest hit by the pandemic, with very high infection and mortality rates in the early pandemic waves. Many such infections have resulted from a combination of factors, including a lack of adequate personal protective equipment (PPE) and infection control guidance in such settings, as well as inconsistent, pre-discharge testing of residents returning from hospital, while they were still infectious and shedding the virus [1,18–20].

Outbreaks of COVID-19 in hospices for palliative care patients (i.e. those expected to die shortly) pose a unique and difficult situation—and often include staff, relatives and patients as possible sources and recipients of infection. Generally, such patients will not be considered for intensive care (i.e. admission to ICU—including intubation and ventilation) or for resuscitation, yet there is an emphasis on maintaining a reasonable quality of life, free from pain, anxiety and distress. In the context of an ongoing pandemic, when staff and laboratory resources may be redirected to more acute care settings, and where visitors are restricted or banned, maintaining such quality end-of-life care can be a challenge [21–24].

Here we present an outbreak of COVID-19 in a hospice setting, during 7 April–6 June 2020, during the first pandemic wave in the UK, when national daily COVID-19 case numbers ranged from 2000 to 5000 per day, and the cumulative numbers increased from 50 000 to 300 000.

## 2. Methods

### 2.1. Hospice setting

The hospice layout was based on a circular plan with the buildings surrounding a central courtyard or garden. Patient facilities mainly consisted of three open bays (A, B, C) containing four to five beds each, with shared bathroom facilities outside of the bay; and 19 single-bedded side-rooms with en-suite bathroom facilities. None of these patient areas had a negative pressure containment capability.

The hospice had a mixture of semi-natural (i.e. no mechanical exhaust, but where the excess supply air flowed out through gaps under the doors) and mechanical ventilation systems, with a rooftop HVAC (heating, ventilation, air-conditioning) system providing a nominal ceiling supply mainly for thermal comfort, with only the bathrooms containing ceiling exhaust vents.

**Figure 1.**
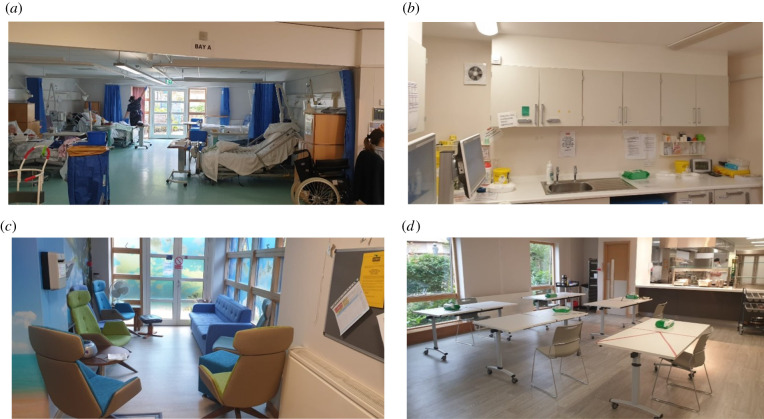
Images of: (*a*) a five-bedded patient bay; (*b*) a treatment room with wall-mounted extractor fan; (*c*) staff break room; (*d*) staff dining room. All of these areas have openable windows that were kept open after aerosol infection control measures were implemented.

There were also many windows in the corridors, bays, side-rooms, administration offices and in the spacious staff cafeteria that could be opened (figure 1). However, not all the doctors' offices contained windows. No attention was given to opening windows beyond managing temperature in the hospice during the early outbreak period and so many windows remained closed. All the windows had latches on them to limit the degree of opening, so their opening was not considered to be a security risk.

In addition, there were multiple small treatment rooms containing drugs, blood- and other sampling equipment and sterile dressings (figure 1). Some of these had wall extractor fans installed, though these were not always operating. None of the treatment rooms had windows.

### 2.2. Patients

There were approximately 10–20 in-patients at any time during this outbreak period (7 April–6 June 2020). The diagnoses of these patients included end-stage cancer, heart and respiratory failure and degenerative neurological diseases.

All patients had a daily medical review on weekdays and as needed at weekends. Most residents required some degree of staff assistance with the activities of daily living, so close contact between staff and patients was common and sometimes prolonged, allowing potential virus transmission.

Ongoing, routine clinical records were maintained by the hospice care team, indicating the date of illness onset, any symptoms and a laboratory-based confirmation of their SARS-CoV-2 infection.

Patients arriving back from any hospital admission, who were sufficiently clinically stable, but with a known positive COVID-19 status, were admitted directly into a single-bedded side room.

Any patients who developed symptoms on open bays were moved to side rooms, pending any repeat testing for COVID-19. For confirmed cases with COVID-19, clinical care included antibiotics if indicated, but no assisted ventilation.

At this early stage of the pandemic, there were no specific antivirals or proven treatments for SARS-CoV-2, and hospice care patients were not eligible for ICU care.

### 2.3. Staff

There were 10–20 ward staff on-site at any time during the outbreak period, consisting of senior and junior doctors, nurses, healthcare assistants (HCAs), physiotherapists and those in non-patient-facing roles in administration, catering, cleaning and estates.

Clinical staff worked on an 8–12 h shift system, with five doctors (two seniors and three juniors), six nurses (one sister, five staff nurses), six HCAs and one physiotherapist on the day shift from 08.00 to 18.00, and four nurses, four HCAs on site and two doctors on-call from home during the evening/overnight shift.

At any time-point, on average, each nurse was caring for about one-third of all patients, with doctors attending to about one-half of all the patients, as there were more nurses than doctors at any one time present on the wards.

### 2.4. Testing

Patient and staff screening for de novo COVID-19 infections were initially focused on symptomatic cases (fever, sore throat, cough, dyspnoea, loss of taste and smell, etc.), but later also included asymptomatic screening of both staff and patients, as ongoing studies demonstrated that a variable proportion of SARS-CoV-2 infections might be asymptomatic [1,25,26].

Nasal and/or throat swabs were taken and sent to the local hospital laboratory for testing using a variety of reverse-transcription polymerase chain reaction (RT-PCR) assays, including: the AusDiagnostics SARS-CoV-2 PCR assay (Ausdiagnostics UK Ltd., Chesham, England), which targeted the SARS-CoV-2 ORF1ab and ORF8 genes [27]; and the RealStar SARS-CoV-2 RT-PCR kit (Altona Diagnostics GmbH, Hamburg, Germany), which targets the SARS-CoV-2 envelope (E) and the glycosylated spike (S) [28].

No SARS-CoV-2 ‘bedside’ point-of-care test was available at this time, so there was a variable delay of approximately 1–3 days before results were reported, depending on the level of sample backlog in the laboratory. No SARS-CoV-2 antibody testing was available at this time.

### 2.5. Personal protective equipment use

Although UK government guidance on social distancing was published on 23 March to coincide with the UK's first national lockdown [29], masking in healthcare setting did not become compulsory until 15 June 2020 in the UK [30].

Prior to this date and during the outbreak period, hospice staff were not masking universally, and the only aerosol-generating procedures were patients using non-invasive ventilation for neurological muscle weakness and occasional NG tube placement—though there was some evidence at that time that oxygen mask and nebulizer use could also spread aerosols [31–33]. Some attempt to mask symptomatic patients was made during April, but this was not tolerated by the patients and was stopped.

So during the outbreak period, staff mask use (type IIR fluid resistant surgical masks) was only for known or suspected cases. Other forms of PPE, such as aprons (but not long-sleeved gowns), gloves, eye protection were only used as normal during specific procedures (e.g. urethral catheterization, wound-dressing).

## 3. Results

### 3.1. Outbreak epidemiology

The admission rate for patients was about 1–2 per day. All but one of the patients who contracted COVID-19 during this period died—deaths that were considered to have been accelerated by COVID-19. Most patients were bed-bound due to their terminal condition, though the first positive case in April was able to move around the bay, which may have contributed to the spread of the virus. Mobility was only restricted for symptomatic patients, who were moved into side rooms.

**Figure 2.**
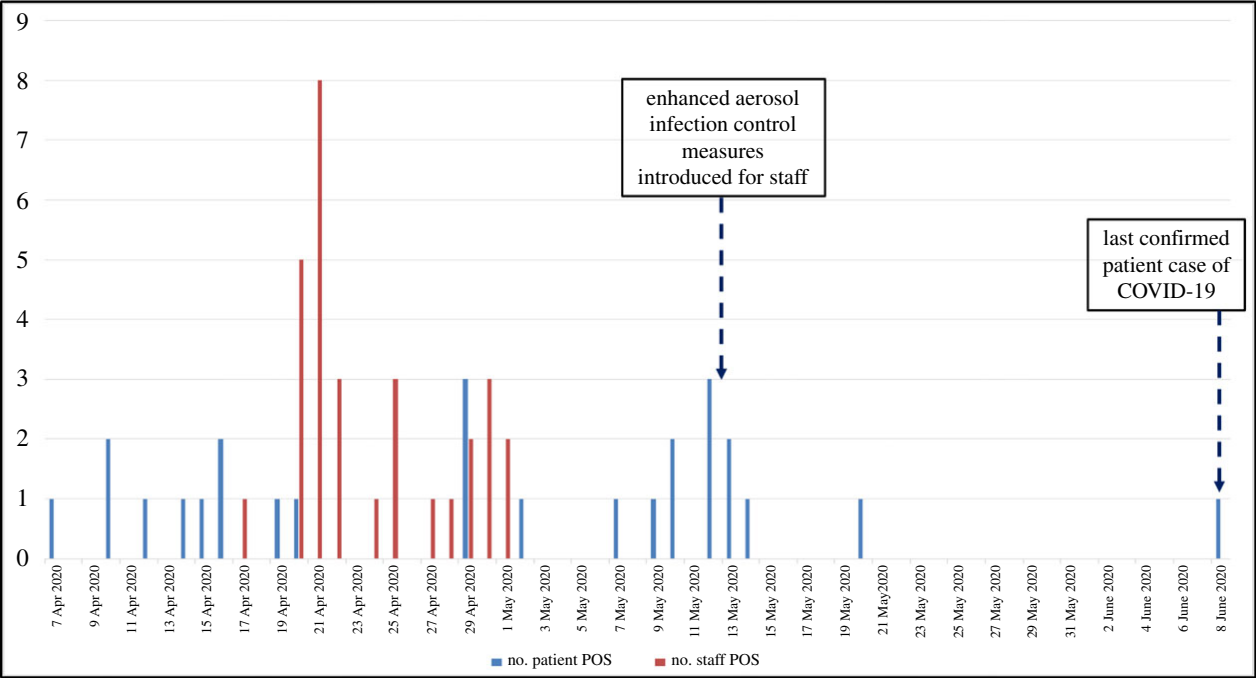
Epidemic curve of daily case numbers during the hospice outbreak showing PCR-confirmed cases in staff and patients, during April–June 2020.

During this outbreak period, 26 patients and 30 staff were identified as SARS-CoV-2 positive by laboratory-based RT-PCR testing, as shown in the epidemic curve (figure 2).

Most infected staff exhibited some symptoms but these were very non-specific and many were relatively mild, so affected staff members may not have voluntarily self-isolated or had themselves tested on this basis. This likely contributed to the long duration of the outbreak (two months).

Similarly, for infected patients, most became symptomatic and were then isolated. There was the potential for some pre-symptomatic patients to act as sources of the virus for the unmasked staff caring for them. Infected staff could then pass the virus onto other patients as they became infected themselves. For most hospice patients there was limited mixing, though at least one patient with COVID-19 did mix with patients in a bay.

### 3.2. Additional infection control advice

In the light of the evolving outbreak, and following a consultation of the clinical lead (L.F.), the duty virologist (J.W.T.) visited the hospice and made the following recommendations:

(i) Due to rapidly accumulating evidence that SARS-CoV-2 was transmitted via aerosols, all available windows on wards, offices, corridors and other common areas—including the cafeteria, should be opened and kept open for as long as possible, to improve the fresh air ventilation in the facility. This would reduce the overall airborne concentration of the virus that might be driving the outbreak.
(ii) All staff should wear surgical masks (Type IIR fluid resistant) throughout their shift, on both the wards as well as in offices and common areas (i.e. non-patient areas), until they leave the hospice to go home. All staff on coffee or lunch breaks, when masks have to be removed, should be taken alone, outdoors or while maintaining at least a 2 m minimal distance apart in well-ventilated areas (e.g. the cafeteria with open windows).
(iii) An inspection of the facility's mechanical ventilation system, with the estates team, in different areas of the hospice was undertaken. From this, it was unclear whether the HVAC system was working as specified, so a ventilation assessment was to be booked for later on in the year. It was further advised that treatment rooms with extractor fans installed should keep them on continuously to improve room ventilation, especially when multiple staff were inside.
(iv) Screening of asymptomatic staff (limited to 5–10 samples a day) was instigated—to detect any potential additional sources of the virus. This testing allocation was limited by diagnostic laboratory capacity, but with an allocation of 10–15 samples a day, 7 days a week, this was able to screen all staff (approx. 30) and patients (approx. 20) every 3–5 days (i.e. at least once a week), which was sufficient to detect any new COVID-19 cases, given an average incubation period of 5–7 days for COVID-19. Infected patients (if otherwise clinically stable) could then be moved to a side-room. Infected staff would be sent home to self-isolate for 14 days (as per guidance at this time).

The clinical lead (L.F.) felt that these measures were convincing and would be potentially effective in terminating this outbreak, to limit any further infections in the staff and patients.

After these measures were instigated, the numbers of COVID-19 cases decreased to zero over the following three weeks (figure 2). As most patients were bed-bound and immobile, and so could not mix with each other, and no visitors were allowed, the patients would have most likely acquired their infection from the staff, who were mobile. So if these infection control interventions were effective, this would be reflected in the staff infection rates first—rather than the patients, of which there were relatively few. This can be seen to some extent in the epidemic curve for staff towards the end of April 2020 (figure 2). Part of this reduction could also have been due to the overall decrease in national COVID-19 incidence in response to the national lockdown (lasting from 23 March to 1 June 2020) [34,35].

### 3.3. Ventilation assessment

The ventilation survey (EMMCOMM Commissioning Ltd, Lincoln, UK) took place in December 2020, about six months after the end of the outbreak. Delays were likely due to competing priorities, ongoing COVID-19 restrictions together with a backlog of work.

The assessment showed that overall, the mechanical ventilation, at the time of the survey (December 2020), was functioning as originally intended, which would have been mainly for thermal comfort (electronic supplementary material, table S1). Yet these ventilation rates fell below the general clinical ward requirements of six air changes per hour (ACH) recommended by UK hospital building pre- and post-COVID-19 standards [36]. The ventilation rates also fell far short of the ideal when occupancy was considered (of at least 10 l s^−1^ per person) in the bays, where there could be 4–5 patients with 2–3 staff at any one time [37].

Given the ventilation rates for bays A, B and C (electronic supplementary material, table S1), for an occupancy of five people (three patients and two staff) in each of the bays A, B and C, the ventilation rates would be approximately: 1.2, 0.62 and 0.87 l s^−1^ per person, respectively. There is evidence that ventilation rates as low as 1–3 l s^−1^ per person can allow super-spreading events [37].

Given the variable and ever-changing levels of staffing and patient occupancy within the bays and rooms, it was difficult to continuously quantify the ventilation rates per person in real time. However, universal staff masking and the opening of windows very likely helped to reduce the overall airborne exposure to SARS-CoV-2, and therefore the spread of nosocomial virus spread.

## 4. Discussion

This outbreak study is an early demonstration that an accurate understanding of the routes of infection, as well as the nature of symptomatic versus asymptomatic infection and transmission, is crucial for controlling its spread [38]. It also highlighted the inadequate ventilation in the facility, which was below the recommended rate in UK healthcare building guidance of six ACH (or 10 l s^−1^ per person)—which may not be uncommon in healthcare facilities elsewhere in the UK [39]. Problems with enhancing ventilation by opening windows, particularly during the colder seasons, have been raised. One option to mitigate this is to bring in additional heaters to allow some window opening to be maintained.

One notable aspect of this study population was that in the early stages of the pandemic, suspected acute COVID-19 cases were generally admitted to hospital, whereas patients admitted to the hospice during this time were for presumed non-COVID-19 related reasons. Thus there was a general lack of consideration that such hospice patients may also have had SARS-CoV-2 infection as a cause for any of their symptoms. This meant that the index of suspicion of COVID-19 in these hospice patients was generally low, and there was little in the way of infection control precautions when managing them upon and during their admission. This led to unexpected and uncontrolled outbreaks in such facilities during this first pandemic wave—as also documented elsewhere [1,18–20].

In addition, ethical issues for palliative care patients and their carers during the pandemic included the willingness of patients to be admitted for inpatient care knowing that there would be no visitors during that stage of the pandemic, to protect both the staff and patients. This inevitably meant that some patients would die from their terminal illness without family around them, which created understandable moral distress for both the staff and patients. The converse of this was also true, where COVID-19 cases in the hospice also posed an infection risk to family members who were similarly distressed at not being able to be with their loved ones in the hospice at their time of death. Responding to these challenges, as the pandemic has progressed, visiting restrictions have been reviewed so that there is now daily visiting for all patients at the hospice unless they have Covid, for whom a single ‘final' visit is allowed if they are not expected to survive the isolation period.

The lack of understanding of these issues in the early stages of the COVID-19 pandemic led to widespread confusion about these particular aspects of SARS-CoV-2, with western countries and the World Health Organization debating the effectiveness of masks, and the importance of asymptomatic infections for the spread of the virus [40–42].

This in turn led to a lack of focus, prioritization and resourcing for the PPE provided to healthcare staff, as well as to diagnostic laboratories for the screening of asymptomatic contacts (in both community and healthcare settings) [43]. As a result, there were serious delays in implementing universal masking for healthcare workers [44], and an unfortunately late recognition that universal screening for COVID-19 in all patients admitted to hospital was necessary to reduce nosocomial infections in both patients and healthcare workers [45].

Although such universal screening upon admission was eventually implemented in UK hospitals at the end of the first wave, soon after this hospice outbreak in June 2020 [46], it is now accepted that the morbidity and mortality arising from the lack of testing for asymptomatic infections, during this first wave, was significant, particularly in elderly care facilities [1,18–20].

Despite difficulties in determining exactly where a SARS-CoV-2 infection has been acquired (even with viral sequencing), increasing experience and knowledge about how a novel pathogen transmits and clinically presents should be applied in real time to our screening and infection control strategies. In fact, there was evidence that SARS-CoV-2 was an airborne infection and caused a proportion of asymptomatic infections (particularly in children) in some earlier reports of COVID-19 clusters and outbreaks in China [2,47].

## 5. Conclusion

Hospices pose some unique issues for an evolving pandemic. Patients in these facilities are usually admitted with an expectation of a short lifespan, with no ICU or resuscitation requirement, yet they are required to be protected from infections (including those nosocomially acquired from staff) and other ailments that may adversely affect the quality of their life that remains.

The early stages of the COVID-19 pandemic focused on hospitalized acute infections and their complications. There was initially much less attention paid to residents and patients in long-term elderly care facilities and hospices, who did not always present COVID-19-like symptoms requiring urgent confirmatory testing and/or hospital admission. Yet, such undiagnosed or unconfirmed cases could then continue to spread the virus to others in their care facility, or to staff and visitors, who might spread the virus further and/or be more severely affected.

Lessons learned in this pandemic should be applied to the next one, including the longitudinal and universal testing of symptomatic cases and their contacts to determine the asymptomatic proportion of those infected. This will help us understand better the nature (including the likely mode(s) of transmission) of the infection and to implement appropriate interventions in a more robust and timely manner.

## Data accessibility

Data are available within the paper and the electronic supplementary material.

The data are provided in electronic supplementary material [48].

## References

[1] Arons MM et al. 2020 Presymptomatic SARS-CoV-2 infections and transmission in a skilled nursing facility. N. Engl. J. Med. **382**, 2081-2090. (10.1056/NEJMoa2008457) 32329971 PMC7200056

[2] Shen Y et al. 2020 Community outbreak investigation of SARS-CoV-2 transmission among bus riders in Eastern China. JAMA Intern. Med. **180**, 1665-1671. (10.1001/jamainternmed.2020.5225) 32870239 PMC7489377

[3] Choi EM, Chu DKW, Cheng PKC, Tsang DNC, Peiris M, Bausch DG, Poon LLM, Watson-Jones D. 2020 In-flight transmission of SARS-CoV-2. Emerg. Infect. Dis. **26**, 2713-2716. (10.3201/eid2611.203254) 32946370 PMC7588512

[4] Park SY et al. 2020 Coronavirus disease outbreak in call center, South Korea. Emerg. Infect. Dis. **26**, 1666-1670. (10.3201/eid2608.201274) 32324530 PMC7392450

[5] Gold JAW et al. 2021 Clusters of SARS-CoV-2 infection among elementary school educators and students in one school district—Georgia, December 2020-January 2021. Morb. Mortal. Wkly. Rep. **70**, 289-292. (10.15585/mmwr.mm7008e4) PMC8344983 33630823

[6] Stein-Zamir C, Abramson N, Shoob H, Libal E, Bitan M, Cardash T, Cayam R, Miskin I. 2020 A large COVID-19 outbreak in a high school 10 days after schools' reopening, Israel, May 2020. Euro Surveill. **25**, 2001352. (10.2807/1560-7917.ES.2020.25.29.2001352) PMC7384285 32720636

[7] Lee JY et al. 2020 Epidemiological and clinical characteristics of coronavirus disease 2019 in Daegu, South Korea. Int. J. Infect. Dis. **98**, 462-466. (10.1016/j.ijid.2020.07.017) 32702415 PMC7371586

[8] Katelaris AL, Wells J, Clark P, Norton S, Rockett R, Arnott A, Sintchenko V, Corbett S, Bag SK. 2021 Epidemiologic evidence for airborne transmission of SARS-CoV-2 during church singing, Australia, 2020. Emerg. Infect. Dis. **27**, 1677-1680. (10.3201/eid2706.210465) 33818372 PMC8153858

[9] Lendacki FR, Teran RA, Gretsch S, Fricchione MJ, Kerins JL. 2021 COVID-19 outbreak among attendees of an exercise facility—Chicago, Illinois, August-September 2020. Morb. Mortal. Wkly. Rep. **70**, 321-325. (10.15585/mmwr.mm7009e2) PMC7948936 33661859

[10] Chu DKW et al. 2021 SARS-CoV-2 superspread in fitness center, Hong Kong, China, March 2021. Emerg. Infect. Dis. **27**, 2230-2232. (10.3201/eid2708.210833) 34004137 PMC8314845

[11] Gu Y, Lu J, Su W, Liu Y, Xie C, Yuan J. 2021 Transmission of SARS-CoV-2 in the karaoke room: an outbreak of COVID-19 in Guangzhou, China, 2020. J. Epidemiol. Glob. Health **11**, 6-9. (10.2991/jegh.k.201007.001) 33095983 PMC7958275

[12] Qian H, Miao T, Liu L, Zheng X, Luo D, Li Y. 2021 Indoor transmission of SARS-CoV-2. Indoor Air **31**, 639-645. (10.1111/ina.12766) 33131151

[13] Tang JW, Marr LC, Li Y, Dancer SJ. 2021 COVID-19 has redefined airborne transmission. BMJ **373**, n913. (10.1136/bmj.n913) 33853842

[14] Tang JW et al. 2021 Dismantling myths on the airborne transmission of severe acute respiratory syndrome coronavirus-2 (SARS-CoV-2). J. Hosp. Infect. **110**, 89-96. (10.1016/j.jhin.2020.12.022) 33453351 PMC7805396

[15] Greenhalgh T, Jimenez JL, Prather KA, Tufekci Z, Fisman D, Schooley R. 2021 Ten scientific reasons in support of airborne transmission of SARS-CoV-2. Lancet **397**, 1603-1605. (10.1016/S0140-6736(21)00869-2) 33865497 PMC8049599

[16] US CDC. 2021 How COVID-19 spreads. Updated 14 July 2021. https://www.cdc.gov/coronavirus/2019-ncov/prevent-getting-sick/how-covid-spreads.html (accessed 4 August 2021).

[17] WHO. 2021 How does COVID-19 spread between people? Updated 30 April 2021. https://www.who.int/news-room/q-a-detail/coronavirus-disease-covid-19-how-is-it-transmitted (accessed 4 August 2021).

[18] White EM, Santostefano CM, Feifer RA, Kosar CM, Blackman C, Gravenstein S, Mor V. 2020 Asymptomatic and presymptomatic severe acute respiratory syndrome coronavirus 2 infection rates in a multistate sample of skilled nursing facilities. JAMA Intern. Med. **180**, 1709-1711. (10.1001/jamainternmed.2020.5664) 33074318 PMC7573793

[19] Panagiotou OA et al. 2021 Risk factors associated with all-cause 30-day mortality in nursing home residents with COVID-19. JAMA Intern. Med. **181**, 439-448. (10.1001/jamainternmed.2020.7968) 33394006 PMC7783593

[20] Dutey-Magni PF, Williams H, Jhass A, Rait G, Lorencatto F, Hemingway H, Hayward A, Shallcross L. 2021 COVID-19 infection and attributable mortality in UK care homes: cohort study using active surveillance and electronic records (March-June 2020). Age Ageing **50**, 1019-1028. (10.1093/ageing/afab060) 33710281 PMC7989651

[21] Abbott J, Johnson D, Wynia M. 2020 Ensuring adequate palliative and hospice care during COVID-19 surges. JAMA **324**, 1393-1394. (10.1001/jama.2020.16843) 32955547

[22] Edmonds PM, Sleeman KE, Lovell N, Chester R, Towers RP, Marshall SA, Higginson IJ, Bajwah S, Prentice W. 2021 The impact of and response to the COVID-19 pandemic on a hospital palliative care team. Future Healthc. J. **8**, 62-64. (10.7861/fhj.2020-0131) 33791463 PMC8004328

[23] Fadul N, Elsayem AF, Bruera E. 2021 Integration of palliative care into COVID-19 pandemic planning. BMJ Support. Palliat. Care. **11**, 40-44. (10.1136/bmjspcare-2020-002364) 32527790

[24] Parekh de Campos A, Daniels S. 2021 Ethical implications of COVID-19: palliative care, public health, and long-term care facilities. J. Hosp. Palliat. Nurs. **23**, 120-127. (10.1097/NJH.0000000000000735) 33633091

[25] Oran DP, Topol EJ. 2020 Prevalence of asymptomatic SARS-CoV-2 infection: a narrative review. Ann. Intern. Med. **173**, 362-367. (10.7326/M20-3012) 32491919 PMC7281624

[26] Johansson MA, Quandelacy TM, Kada S, Prasad PV, Steele M, Brooks JT, Slayton RB, Biggerstaff M, Butler JC. 2021 SARS-CoV-2 transmission from people without COVID-19 symptoms. JAMA Netw. Open **4**, e2035057. (10.1001/jamanetworkopen.2020.35057) 33410879 PMC7791354

[27] Attwood LO, Francis MJ, Hamblin J, Korman TM, Druce J, Graham M. 2020 Clinical evaluation of AusDiagnostics SARS-CoV-2 multiplex tandem PCR assay. J. Clin. Virol. **128**, 104448. (10.1016/j.jcv.2020.104448) 32460173 PMC7236671

[28] Visseaux B et al. 2020 Evaluation of the RealStar® SARS-CoV-2 RT-PCR kit RUO performances and limit of detection. J. Clin. Virol. **129**, 104520. (10.1016/j.jcv.2020.104520) 32652476 PMC7323686

[29] Public Health England. 2020 Coronavirus (COVID-19). Keeping away from other people: new rules to follow from 23 March 2020. https://assets.publishing.service.gov.uk/government/uploads/system/uploads/attachment_data/file/876699/COVID-19_Keeping_away_from_other_people_20200328.pdf (accessed 4 August 2021).

[30] UK Department of Health and Social Care. 2020 Face masks and coverings to be worn by all NHS hospital staff and visitors. 5 June 2020. https://www.gov.uk/government/news/face-masks-and-coverings-to-be-worn-by-all-nhs-hospital-staff-and-visitors (accessed 4 August 2021).

[31] Ferioli M, Cisternino C, Leo V, Pisani L, Palange P, Nava S. 2020 Protecting healthcare workers from SARS-CoV-2 infection: practical indications. Eur. Respir. Rev. **29**, 200068. (10.1183/16000617.0068-2020) 32248146 PMC7134482

[32] Tang JW, Kalliomaki P, Varila TM, Waris M, Koskela H. 2020 Nebulisers as a potential source of airborne virus. J. Infect. **81**, 647-679. (10.1016/j.jinf.2020.05.025) PMC7227527 32422151

[33] Public Health England. 2021 Guidance. 6. COVID-19 infection prevention and control guidance: aerosol generating procedures—procedures that create a higher risk of respiratory infection transmission. Updated 1 June 2021. https://www.gov.uk/government/publications/wuhan-novel-coronavirus-infection-prevention-and-control/covid-19-infection-prevention-and-control-guidance-aerosol-generating-procedures (accessed 4 August 2021).

[34] Institute for Government UK. 2021 Timeline of UK coronavirus lockdowns, March 2020 to March 2021. https://www.instituteforgovernment.org.uk/sites/default/files/timeline-lockdown-web.pdf.

[35] Worldometer. 2021 Coronavirus cases. United Kingdom. https://www.worldometers.info/coronavirus/country/uk/ (accessed 16 November 2021).

[36] Department of Health, UK. 2021 Health Technical Memorandum 03-01. Specialised ventilation for healthcare premises Part A: The concept, design, specification, installation and acceptance testing of healthcare ventilation systems. Updated 22 June 2021. https://www.england.nhs.uk/wp-content/uploads/2021/05/HTM0301-PartA-accessible-F6.pdf (accessed 4 August 2021).

[37] UK SAGE Environment and Modelling Group. 2021 Simple summary of ventilation actions to mitigate the risk of COVID-19. https://assets.publishing.service.gov.uk/government/uploads/system/uploads/attachment_data/file/945754/S0973_Ventilation_Actions_Summary_16122020_V2.pdf (accessed 4 August 2021).

[38] Fraser C, Riley S, Anderson RM, Ferguson NM. 2004 Factors that make an infectious disease outbreak controllable. Proc. Natl Acad. Sci. USA **101**, 6146-6151. (10.1073/pnas.0307506101) 15071187 PMC395937

[39] UK Department of Health and Social Care. 2021 Build back better: £600 million to upgrade and refurbish NHS hospitals. https://www.gov.uk/government/news/build-back-better-600-million-to-upgrade-and-refurbish-nhs-hospitals (accessed 4 August 2021).

[40] Klompas M, Morris CA, Sinclair J, Pearson M, Shenoy ES. 2020 Universal masking in hospitals in the COVID-19 era. N. Engl. J. Med. **382**, e63. (10.1056/NEJMp2006372) 32237672

[41] Tang JW. 2020 COVID-19: interpreting scientific evidence—uncertainty, confusion and delays. BMC Infect. Dis. **20**, 653. (10.1186/s12879-020-05387-8) 32895050 PMC7476768

[42] Cowling BJ, Leung GM. 2020 Face masks and COVID-19: don't let perfect be the enemy of good. Euro Surveill. **25**, 2001998. (10.2807/1560-7917.ES.2020.25.49.2001998) PMC7730488 33303063

[43] Iacobucci G. 2020 COVID-19: lack of capacity led to halting of community testing in March, admits deputy chief medical officer. BMJ **369**, m1845. (10.1136/bmj.m1845) 32376638

[44] Richterman A, Meyerowitz EA, Cevik M. 2020 Hospital-acquired SARS-CoV-2 infection: lessons for public health. JAMA **324**, 2155-2156. (10.1001/jama.2020.21399) 33185657

[45] Shah ASV et al. 2020 Risk of hospital admission with coronavirus disease 2019 in healthcare workers and their households: nationwide linkage cohort study. BMJ **371**, m3582. (10.1136/bmj.m3582) 33115726 PMC7591828

[46] Public Health England. 2021 COVID-19: guidance for maintaining services within health and care settings. Infection control and prevention recommendations. January 2021. See https://assets.publishing.service.gov.uk/government/uploads/system/uploads/attachment_data/file/954690/Infection_Prevention_and_Control_Guidance_January_2021.pdf (accessed 23 April 2021).

[47] Bai Y, Yao L, Wei T, Tian F, Jin DY, Chen L, Wang M. 2020 Presumed asymptomatic carrier transmission of COVID-19. JAMA **323**, 1406-1407. (10.1001/jama.2020.2565) 32083643 PMC7042844

[48] Feathers L, Hinde T, Bale T, Hyde J, Bird PW, Holmes CW, Tang JW. 2022 Outbreak of SARS-CoV-2 at a hospice: terminated after the implementation of enhanced aerosol infection control measures. Figshare. 10.1098/rsfs.2021.0066 PMC8831080 35261730

